# Strength Prediction Method for Phosphogypsum Concrete Based on Dynamic Weighted Transfer Learning

**DOI:** 10.3390/ma18225206

**Published:** 2025-11-17

**Authors:** Pan Chen, Feng Zhu, Dongxu Zhang, Pengfei Liu, Hongjun Liang

**Affiliations:** 1 School of Civil Engineering, Hubei Engineering University, Xiaogan 432000, China; chenpan@hbeu.edu.cn (P.C.);; 2 Hubei Province Engineering Research Center for Cement-Based Ultra-High Performance Concrete and Prefabricated Building Technology, Xiaogan 432000, China; 3 School of Civil Engineering, Wuhan University, Wuhan 430072, China

**Keywords:** phosphogypsum concrete, strength prediction, transfer learning, data augmentation, small-sample

## Abstract

Recycling industrial solid waste phosphogypsum into phosphogypsum concrete (PGC) is a crucial pathway for achieving high-value solid waste utilization. However, the scarcity of experimental samples for PGC has led to inaccurate predictions of compressive strength by traditional models, severely hindering its application. This study proposes a dynamic weighted transfer learning-based method for predicting the strength of PGC, addressing the characterization bottleneck under small-sample conditions by transferring knowledge from the strength patterns of conventional concrete. First, feature differences between conventional concrete and PGC are eliminated through component proportion normalization and feature alignment. Then, a data augmentation technique based on Bootstrap Resampling is developed to generate enhanced samples that comply with mix proportion constraints, effectively expanding the training samples. Finally, an error feedback-driven dynamic weight calculation and weighted loss optimization framework for transfer learning is designed, prioritizing the learning of samples in the prediction blind spots of the target domain. This enables the adaptive acquisition of PGC-specific knowledge while inheriting the general knowledge of conventional concrete. Experimental results show that the transfer learning model achieves a prediction accuracy of R^2^ = 0.95 on the target domain test samples, a 15.9% improvement over traditional methods, while maintaining robust performance (R^2^ = 0.97) on an external validation samples. Feature importance analysis and Shapley Additive Explanations (SHAP) analysis reveal the nonlinear coupling effects of PGC-specific parameters on strength. This study establishes a scientific approach for accurate strength prediction of PGC under small-sample conditions.

## 1. Introduction

Phosphogypsum, as a major byproduct of the phosphorus fertilizer industry, has an annual global production of approximately 300 million tons, with historical stockpiles exceeding 7 billion tons. Research indicates that properly treated phosphogypsum can replace 50% of cement in concrete production. This waste-as-material application not only alleviates the environmental pressure caused by phosphogypsum stockpiling but also reduces energy consumption and pollution emissions from natural resource extraction and cement production, offering significant value for promoting the green and low-carbon transformation of the construction materials industry [1,2,3]. However, in the mix design process of phosphogypsum concrete (PGC), the prediction of compressive strength faces dual challenges: scarce experimental samples and nonlinear coupling among multiple components. Traditional empirical models struggle to characterize such nonlinear relationships, and while existing machine learning methods have made progress in predicting the strength of conventional concrete, they are often limited by small-sample conditions, leading to model underfitting [4,5]. Therefore, there is an urgent need to develop high-precision methods tailored for predicting the strength of PGC, aiming to overcome data limitations and reveal the synergistic mechanisms among raw material components, thereby providing a reliable tool for intelligent PGC design.

In recent years, numerous scholars have conducted theoretical research on concrete strength prediction. By incorporating fiber reinforcement coefficients, hybrid effect coefficients, and composite mechanics theory, researchers have significantly improved the prediction accuracy and applicability of their models [6,7]. Abdeldjalil et al. [8] combined fractal dimension and water-cement ratio to develop an exponential prediction equation, achieving an R^2^ = 0.99 for specific datasets and revealing the synergistic effect between particle gradation and hydration reactions. Moutassem & Kharseh [9] proposed a multi-parameter strength prediction model for silica fume concrete, integrating hydration degree and packing density, though temperature nonlinearities were not discussed. Li et al. [10] introduced the water-cement density ratio law, integrating relative apparent density into strength prediction and unifying four heterogeneous materials. However, their linear superposition assumption fails to explain nonlinear interactions between materials. Gao et al. [7] developed a prediction model based on composite mechanics theory, incorporating fiber reinforcement coefficients and synergistic effect coefficients, demonstrating that a combination of 0.2% polypropylene fiber and 1.5% steel fiber yields optimal strengthening efficiency. Although existing research has made significant progress in theoretical modeling for concrete strength prediction, further breakthroughs are needed to move beyond the limitations of linear superposition assumptions and explore the nonlinear coupling mechanisms in multi-material systems more deeply. This will enable more scientific and universally applicable strength prediction methods.

Machine learning techniques, through data-driven modeling, offer a novel approach to strength prediction, demonstrating significant advantages in handling multi-factor nonlinear relationships and reducing experimental costs [11]. El-Mir et al. [12] systematically compared Gaussian process regression (GPR), support vector machine (SVM), and regression tree models, finding that GPR achieved a root mean square error (RMSE) as low as 3.09 MPa for ultra-high-performance concrete, highlighting the impact of algorithm selection on prediction accuracy. Gupta [13] employed artificial neural network (ANN) to develop a geopolymer concrete prediction model, achieving an R^2^ of 0.904, which underscores the advantages of nonlinear activation functions. Güçlüer et al. [14,15] evaluated the performance of decision trees, SVM, and ANN, confirming that decision trees yielded a mean absolute error (MAE) of only 2.59 MPa under humidity influence but lacked sensitivity to high-dimensional features. Li et al. [16] applied Random Forest (RF) to basalt fiber-reinforced concrete, with confining pressure parameters scoring 0.42 in feature importance yet failing to resolve the instability in interpretability caused by feature interaction effects. Collectively, these studies indicate that the choice of machine learning models and feature engineering directly affects prediction accuracy. However, insufficient analysis of parameter interaction effects leads to significant instability in feature importance determination.

With the deepening application of machine learning in concrete strength prediction, researchers have begun exploring more advanced neural network architectures and ensemble strategies to further enhance models’ ability to characterize complex nonlinear relationships. Deep learning methods, through multi-level feature abstraction and nonlinear transformations, provide new technical pathways to overcome the performance bottlenecks of traditional machine learning algorithms. Aggarwal et al. [17] introduced deep neural networks into self-compacting concrete prediction, achieving a correlation coefficient of 0.907 using ReLU activation functions—a 3% improvement over traditional ANN. Li et al. [18] developed a stacked ensemble model combining the strengths of RF and eXtreme Gradient Boosting (XGBoost), achieving an R^2^ of 0.987 for rice husk ash concrete, though it did not resolve the underfitting risk under small-sample conditions. Zhang et al. [19] constructed a deep forest model that captured slag-superplasticizer interactions through multi-granularity scanning, reducing test set errors by 18% compared to single tree models. Mahmood et al. [20] compared eight algorithms for marble powder-modified concrete and found that ensemble models achieved an R^2^ exceeding 0.998 but excessive reliance on ideal mix-proportion data led to conflicts with physical constraints. These cases demonstrate that while increased algorithmic complexity can significantly enhance predictive capabilities for complex material systems, over-reliance on data fitting while neglecting deeper material science mechanisms results in severe robustness challenges when facing data noise and physical constraints.

Transfer learning offers an innovative approach to resolving the inherent contradictions between traditional machine learning and deep learning methods in concrete strength prediction. Faced with challenges such as unstable feature interaction analysis under small-sample conditions and physical constraint conflicts caused by complex models’ over-reliance on data fitting, transfer learning achieves dual breakthroughs through cross-material domain knowledge transfer mechanisms [21,22,23]. The core idea lies in the collaborative modeling of general patterns established in the source domain and specific patterns in the target domain. First, by mapping prior knowledge from the source domain (such as water-cement ratio laws and aggregate gradation effects) to the target domain, the stability trained from the source domain’s large data compensates for the volatility of feature analysis in the target domain’s small-sample scenarios, thereby mitigating the instability of feature importance determination. Second, dynamic domain adaptation strategies are employed to inherit general physical constraints from the source domain during knowledge transfer, preventing deviations from physicochemical laws caused by purely data-driven approaches in deep learning. This framework not only enhances the robustness of small-sample predictions but also ensures that model outputs remain consistent with fundamental material science principles.

In the application of transfer learning to concrete strength prediction, scholars have achieved a series of significant advancements. Ford et al. [24] developed a transfer learning framework combined with empirical formula-based data augmentation, enabling cross-property prediction from compressive strength to elastic modulus, with results showing R^2^ = 0.858. However, their reliance on American Concrete Institute 318 (ACI 318) empirical relationships may introduce model bias. Nguyen et al. [25] created a convolutional neural network-TabNet (CNN-TabNet) hybrid model that leveraged conventional geopolymer data to enhance prediction accuracy for ultra-high-performance variants, reducing RMSE by 30%, though it failed to address missing features such as aggregate morphology. Zhang et al. [26] innovatively established a transfer learning model for alkali-activated slag-based foam concrete, achieving target domain prediction errors within ±10% through source domain pretraining, but their data scope was limited to laboratory-standard mix proportions. While these studies have demonstrated the effectiveness of transfer learning in concrete strength prediction, no existing research has explored its application to small-sample PGC strength prediction.

Despite significant progress in conventional concrete strength prediction methods, existing research faces the following challenges when applied to PGC. First, small-sample enhancement techniques face adaptability bottlenecks. Traditional oversampling methods generate pseudo-samples that often violate material component conservation laws [27], leading to samples that conflict with fundamental material science principles. Second, static weight allocation strategies struggle to capture target-domain-specific patterns. Conventional transfer learning employs fixed weights for cross-domain knowledge transfer, lacking an error-feedback-driven dynamic adjustment mechanism. This approach neither identifies the nonlinear coupling effects unique to phosphogypsum characteristics nor focuses on prediction blind spots in the target domain under limited sample conditions. These limitations collectively lead to inefficient cross-domain knowledge transfer and inadequate small-sample generalization capability in existing models for PGC strength prediction, necessitating the development of novel transfer learning models.

Therefore, a significant research gap exists in the absence of a high-precision, robust prediction model for PGC compressive strength, particularly under the practical constraint of small sample sizes. While transfer learning has shown promise in other concrete types, its specific application and optimization for PGC, which has unique material components and nonlinearities, remains unexplored. The primary purpose of this study is to bridge this gap by developing and validating a novel dynamic weighted transfer learning framework. This framework is specifically designed to leverage the vast knowledge from conventional concrete to accurately predict the strength of PGC even with limited experimental data, thereby providing a reliable tool for intelligent PGC design and promoting the sustainable utilization of this industrial byproduct.

This study proposes a dynamic weighted transfer learning-based method for PGC strength prediction. First, data collection and preprocessing are performed, where component proportion normalization and feature alignment eliminate feature disparities between conventional concrete and PGC. Next, the Bootstrap Resampling data augmentation method is used to effectively expand target-domain samples while maintaining mix proportion constraints. Finally, model training is conducted within a dynamic weighted transfer learning algorithmic framework, producing evaluation metrics and visualizations. During the model validation, performance differences between traditional machine learning benchmark models and the transfer learning model are comparatively analyzed, and an external validation set is used to assess the transfer learning model’s generalization capability.

## 2. Methodology

### 2.1. Bootstrap Resampling

**Figure 1:**
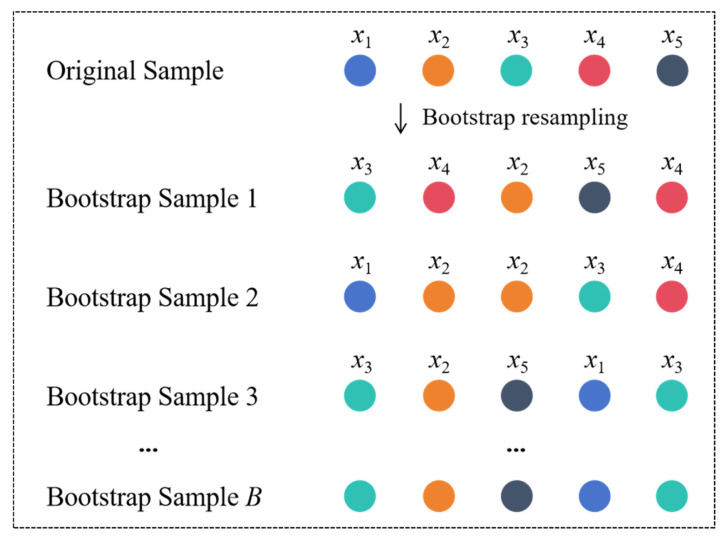
Principle of bootstrap resampling.

Bootstrap Resampling is a statistical inference method based on empirical distribution, originally proposed by Efron [28]. Its fundamental principle involves generating numerous simulated sample sets from the original datasets through repeated sampling with replacement, thereby expanding the data. Figure 1 illustrates its schematic diagram. This method requires no presupposition about data distribution patterns, relying solely on random sampling from original samples, thus providing a theoretical foundation for small-sample data augmentation.

Given an independent and identically distributed datasets *D* = {*x*_1_, *x*_2_, …, *x*_n_} following an unknown distribution *F*, Bootstrap Resampling constructs an empirical distribution function ${\hat{F}}_{n}$ as an approximation of *F*, defined as follows:

(1) $${\hat{F}}_{n}(x)=\frac{1}{n}\sum_{i=1}^{n}I\;\;(x_{i}\leq x)$$

where *I* is the indicator function. Based on ${\hat{F}}_{n}$, *m* samples are drawn with replacement from *D* to generate a bootstrap sample set $D^{*}=\{x_{1}^{*},x_{2}^{*},\ldots ,x_{m}^{*}\}$, whose probability mass function satisfies:

(2) $$P(x^{*}=x_{i})=\frac{1}{n},\;\forall i\in \{1,2,\ldots ,n\}$$

Repeating this process *B* times generates *B* independent bootstrap sample sets $\left\{D^{*1},D^{*2},\ldots ,D^{*B}\right\}$, effectively expanding the data distribution from ${\hat{F}}_{n}$ to ${\hat{F}}_{B}^{n}$. In a single sampling, the probability that any original sample is not selected is: $P_{\mathrm{excl}}={\left(1-1/n\right)}^{n}$, when n→∞, $P_{\mathrm{excl}}\approx 36.8\%$.

This study applies Bootstrap Resampling at the level of entire experimental samples. The method generates new sample sets by resampling complete data points (i.e., entire rows from the original dataset), rather than creating new, synthetic mix proportions by combining components from different original samples. Each data point in a bootstrapped sample is a direct replica of a real, physically validated experiment from the original dataset.

**Figure 2:**
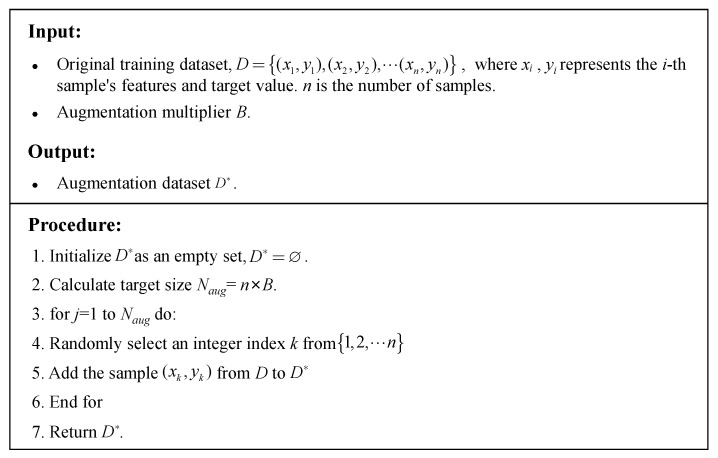
Bootstrap resampling algorithm.

This approach inherently ensures that all generated samples strictly adhere to the fundamental principles of mass conservation and component boundedness. Since no new mix proportions are synthesized, there is no risk of generating physically implausible samples. The primary function of Bootstrap Resampling in this context is to expand the training dataset and mitigate model underfitting by creating diverse training sets, not to invent new material formulations. The precise implementation is detailed in Figure 2. Compared to traditional oversampling methods (e.g., Synthetic Minority Over-sampling Technique, SMOTE), Bootstrap Resampling derives data from real samples, making it particularly suitable for concrete mix proportion data with physicochemical constraints. This avoids generating invalid samples that violate the conservation laws of material composition.

### 2.2. Principles of LightGBM

LightGBM (Light Gradient Boosting Machine) is an efficient gradient boosting framework developed by Microsoft [29]. The algorithm is based on decision trees and features core innovations such as histogram optimization strategies and asymmetric growth mechanisms, making it particularly suitable for modeling high-dimensional data. Compared to traditional gradient boosting trees (GBDT) and algorithms like XGBoost, LightGBM’s theoretical foundation revolves around four key mechanisms: histogram-accelerated algorithm, leaf-wise growth, Gradient-based One-Side Sampling (GOSS), and feature parallelism.

The histogram algorithm discretizes continuous feature values into finite intervals (bins) to construct statistical histograms instead of traversing raw data. This approach pre-splits feature values into *k* bins and accumulates gradient statistics for each bin during training, significantly reducing the complexity of split-point searches. Additionally, LightGBM employs histogram differencing to accelerate computations. The histogram of a parent node can be reconstructed from the difference between its child node histograms, minimizing redundant statistical calculations. Since PGC involves multiple features such as cement, phosphogypsum, and retarders, with complex nonlinear relationships among parameters, the histogram algorithm effectively reduces sensitivity to noise, preventing overfitting.

The leaf-wise growth strategy directly splits the leaf node with the highest gain, enabling more efficient decision tree construction. Unlike traditional level-wise strategies, leaf-wise growth avoids inefficient traversal of all nodes at the same level, focusing instead on locally optimal splitting paths. This significantly improves model convergence speed and prediction accuracy. To prevent overfitting, LightGBM introduces a maximum depth limit to control tree complexity. For small samples, traditional level-wise growth requires traversing all nodes, often leading to ineffective splits. In contrast, the leaf-wise strategy prioritizes splitting nodes with the highest gain, allowing critical features to form decision paths quickly in limited samples, thereby enhancing feature resolution efficiency.

GOSS is an efficient data sampling method for gradient boosting trees. Its core principle leverages the asymmetry of gradient distributions—samples with larger absolute gradients contribute more to information gain. GOSS first sorts samples in descending order of gradient magnitude, retaining the top *a*% of high-gradient samples to ensure accurate information gain calculations. It then randomly samples *b*% of the remaining low-gradient samples to maintain data distribution. To mitigate distribution bias from low-gradient samples, the algorithm applies a compensation factor (1 − *a*)/*b* to their gradients, minimizing information loss while reducing computational complexity. This asymmetric sampling strategy strengthens the model’s focus on prediction-blind samples, improving local fitting capability in transfer learning.

The feature parallelism addresses the communication bottleneck caused by vertical data partitioning in traditional methods. Conventional approaches (e.g., XGBoost) require synchronizing feature split information across machines, incurring high communication overhead. LightGBM, however, retains full data on each machine, allowing independent computation of local optimal split points and only synchronizing split results rather than raw data. This design eliminates inconsistencies in data partitioning and avoids redundant computations for feature index reconstruction, significantly improving training efficiency in large-scale feature scenarios.

### 2.3. Dynamic Weighted Transfer Learning Framework

**Figure 3:**
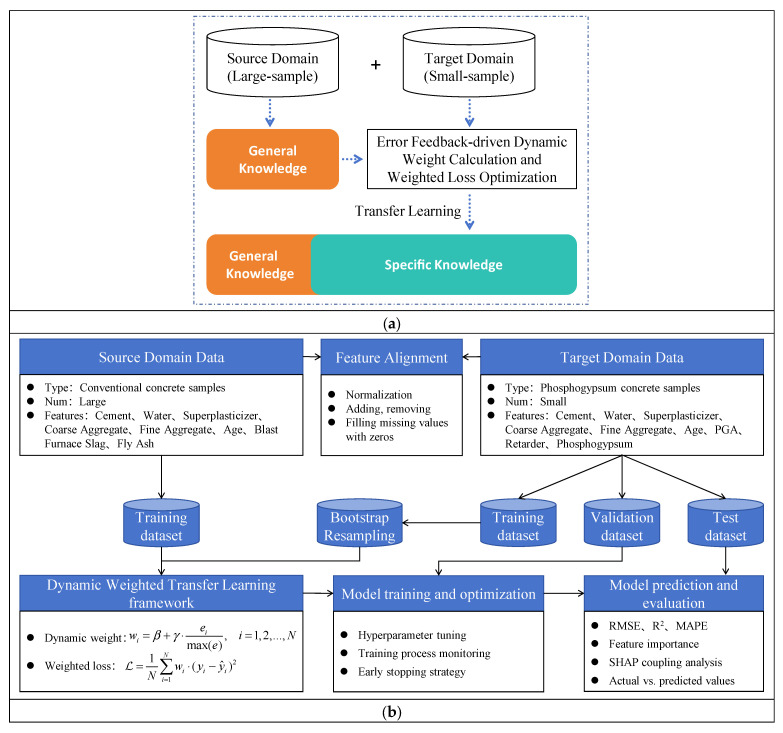
Dynamic weighted transfer learning model. (**a**) Principles of transfer learning; (**b**) Dynamic weighted transfer learning framework.

The proposed dynamic weighted transfer learning framework is illustrated in Figure 3, which mainly consists of four parts: feature alignment, data augmentation, dynamic weight calculation, and weighted loss optimization.

(1) Feature Alignment

Feature space transformation based on material composition conservation laws is established to mitigate the feature differences between the source domain (conventional concrete) and target domain (PGC). This includes component proportion normalization and feature alignment.

Component proportion normalization applies total mass constraint normalization to features in both domains, converting absolute quantities into mass proportions to eliminate formulation scale differences. The calculation formula is:

(3) $$C_{i}^{norm}=\frac{C_{i}}{\sum_{j=1}^{q}\left(C_{j}\right)}$$

where *C_i_* is the absolute dosage (kg/m^3^) of the *i*-th component, and *q* is the number of raw material types. The operation converts each component into a proportion of the total mass (∑ = 1), thereby eliminating absolute dosage differences across formulations.

The age feature is normalized to the range [0, 1] using:

(4) $$Age^{norm}=\frac{Age-Age_{min}}{Age_{max}-Age_{min}}$$

Feature alignment involves adding target-domain-specific feature columns to the source domain data, filling missing values with zeros, while removing source-domain-specific features. A joint feature space containing all target-domain features is constructed, achieving complete dimensional consistency between the source and target domains.

(2) Data Augmentation

Bootstrap Resampling is applied to expand the samples and overcome target-domain sample scarcity. Target-domain training samples are randomly drawn with replacement, generating an augmented sample set *B* times the size of the original samples. The resampling process ensures that newly generated samples strictly adhere to mixture ratio conservation constraints.

(3) Dynamic Weight Calculation

An error feedback-driven sample importance evaluation system is established. A LightGBM baseline model is pre-trained on the original target-domain training samples to obtain the absolute prediction error for each sample:

(5) $$e_{i}=|y_{i}-{\hat{y}}_{i}|$$

where $y_{i}$ and ${\hat{y}}_{i}$ are the true and predicted values, respectively.

Error-driven dynamic weights are assigned to augmented samples using:

(6) $$w_{i}=\beta +\gamma \cdot \frac{e_{i}}{\max(e)},\;i=1,2,\ldots ,N$$

where *w_i_* represents dynamic weight of the *i*-th sample; *β* and *γ* are hyperparameters. Based on a sensitivity analysis detailed in Section 4.5, these were set to *β* = 2 and *γ* = 3 to achieve optimal model performance. max(*e*) is error normalization factor. *N* represents the number of samples. The weighting method ensures that samples with larger errors receive higher weights, guiding the model to prioritize learning prediction-blind spots in the target domain.

(4) Weighted Loss Optimization

An error-weighted mean squared loss function is constructed:

(7) $$L=\frac{1}{N}\sum_{i=1}^{N}w_{i}\cdot {\left(y_{i}-{\hat{y}}_{i}\right)}^{2}$$

During training, the source-domain samples are uniformly assigned a base weight *w* = 1. The target-domain augmented samples receive dynamic weights based on error proportions, as defined in Equation (6). Specifically, the dynamic component of the weights is normalized by dividing the individual sample error *eᵢ* by the maximum error max(*e*) within the original target-domain training set. This ensures the weights for target-domain samples are scaled consistently in a range of [2, 5].

The model training was conducted using the LightGBM framework. The key hyperparameters were carefully selected to balance performance and prevent overfitting. The objective function was set to ‘regression’ with the Mean Squared Error (MSE) as the evaluation metric. Other critical hyperparameters included: a learning_rate of 0.02, num_leaves set to 31, min_child_samples of 10, and a feature_fraction of 0.7. To mitigate overfitting, L_1_ and L_2_ regularization terms were both set to 0.1. The training was configured for a maximum of 1000 boosting rounds, but an early stopping mechanism was implemented. This mechanism monitored the validation loss and terminated the training process if no improvement was observed for 50 consecutive iterations, ensuring the final model did not overfit to the training data.

## 3. Data and Experimental Preparation

### 3.1. Source Domain Data

The source domain data used in this study were obtained from the publicly available concrete compressive strength datasets in the UCI Machine Learning Repository (UC Irvine Machine Learning Repository), comprising a total of 1030 experimental samples (denoted as S1) [30]. This datasets comprehensively cover the key compositional parameters of conventional concrete. The input features include eight critical variables: Cement, Blast Furnace Slag, Fly Ash, Water, Superplasticizer, Coarse Aggregate, Fine Aggregate, and Age. The target output is the Compressive Strength of concrete specimens under standard curing conditions.

The distribution of each component in the datasets exhibits significant diversity, as shown in Table 1. The Cement ranges from 102 to 540 kg/m^3^. Among mineral admixtures, the maximum of Blast Furnace Slag and Fly Ash reach 359.4 kg/m^3^ and 200.1 kg/m^3^, respectively, with minimum values of zero. The Water ranges from 121.8 to 247.0 kg/m^3^, and the Superplasticizer ranges from 0 to 32.2 kg/m^3^. For the Aggregate system, the Coarse and Fine aggregate range from 801.0 to 1145.0 kg/m^3^ and 594.0 to 992.6 kg/m^3^, respectively. The Age parameter spans 1 to 365 days, with a focus on the critical strength development period of 7 to 90 days.

### 3.2. Target Domain Data

The target domain data in this study were derived from compressive strength tests on PGC conducted by the authors’ research team. All specimen preparation and testing procedures strictly adhered to the Chinese standard GB/T 50081-2019 [31]. A total of 19 valid samples (denoted as S2) were obtained. The input features encompass nine key parameters: Cement, Water, Superplasticizer, Coarse Aggregate, Fine Aggregate, Phosphogypsum Aggregate (PGA), Retarder, Phosphogypsum and Age. The target output is the Compressive Strength under standard curing conditions.

In terms of feature parameters, the S2 PGC datasets differs significantly from the S1 conventional concrete datasets (Table 1 vs. Table 2). S1 includes two typical industrial admixtures Blast Furnace Slag and Fly Ash, whereas S2 features Phosphogypsum and PGA as core characteristics, along with the addition of Retarder to regulate setting time. The shared features between the two datasets are Cement, Water, Superplasticizer, Coarse Aggregate, Fine Aggregate, and Age. However, S1 lacks three feature parameters present in S2: Phosphogypsum, PGA and Retarder.

The experimental samples cover the key parameter ranges of PGC, as shown in Table 2. The Cement ranges from 299.8 to 608.4 kg/m^3^, and the Water ranges from 156.0 to 206.9 kg/m^3^. The Superplasticizer ranges from 12.0 to 12.2 kg/m^3^. The Coarse aggregate ranges from 0.0 to 810.0 kg/m^3^, while Fine Aggregate and Retarder are constant at 810.0 kg/m^3^ and 1.2 kg/m^3^, respectively. The phosphogypsum content ranges from 0.0 to 304.2 kg/m^3^. The age parameter spans 3 to 28 days, with a focus on the critical strength development periods of 3 and 7 days.

An external validation datasets S3 was constructed to validate the model’s generalization capability. The datasets were sourced from Gong et al.’s [32] research on PGC, comprising 18 experimental samples. All specimen preparation and testing followed Chinese standards. The input features comprise five parameters: Cement, Water, Fine Aggregate, Phosphogypsum and Age, with Compressive Strength under standard curing conditions as the target output. Compared to S1, the feature dimensionality of S3 is reduced to five, lacking Blast Furnace Slag, Fly Ash, Superplasticizer, and Coarse Aggregate but including the new Phosphogypsum feature.

The parameter distribution of the external validation data S3 is shown in Table 3. The Cement ranges from 45.0 to 450.0 kg/m^3^, while Water and Fine Aggregate are constant at 225.0 kg/m^3^ and 1350 kg/m^3^, respectively. The Phosphogypsum ranges from 0.0 to 405.0 kg/m^3^. The Ages include the critical strength development periods of 3, 7, and 28 days.

### 3.3. Domain Similarity Analysis

A domain similarity analysis was conducted to justify the feasibility of transferring knowledge from the conventional concrete domain S1 to the phosphogypsum concrete domain S2 This step is crucial to ensure that the feature distributions of the source and target domains share sufficient overlap, which is a prerequisite for effective transfer learning. Two methods were employed for this analysis, including comparative distribution for key shared features and t-SNE for visualizing the overall data structure, which are presented collectively in Figure 3.

**Figure 4:**
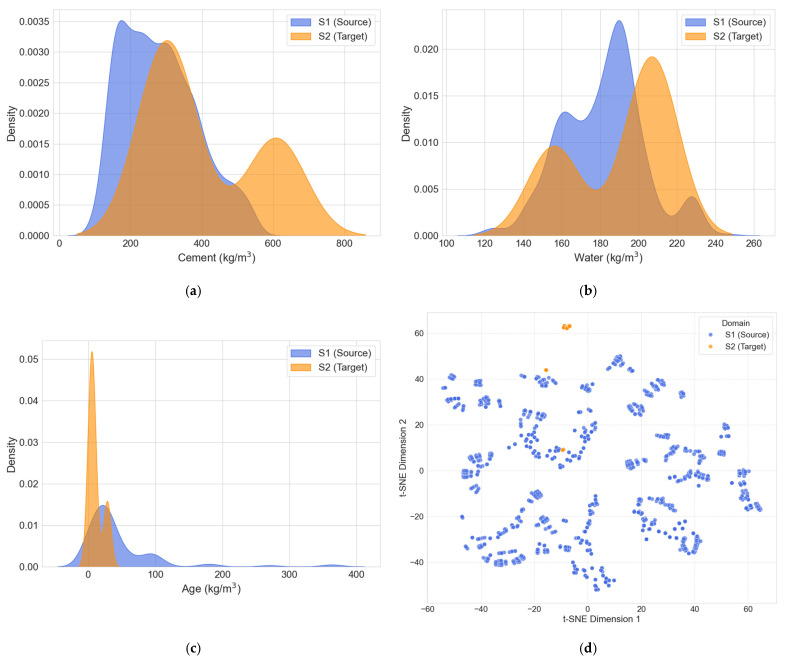
Domain similarity analysis and t-SNE visualization. (**a**) Comparative distributions of Cement; (**b**) Comparative distributions of Water; (**c**) Comparative distributions of Age; (**d**) t-SNE visualization.

Figure 4a–c illustrate the comparative distributions for three principal shared features: Cement, Water, and Age. Other shared features, such as Superplasticizer, Coarse Aggregate, and Fine Aggregate, were not visualized because they exhibited minimal or no variance within the S2 target dataset, rendering a distributional comparison uninformative. The analysis of the visualized features reveals that while the ranges and peaks of the distributions differ, there is a significant overlap. For instance, the cement content in S2 falls largely within the range observed in S1. Similarly, the key parameters of Water and Age show considerable overlap. This shared feature space suggests that the fundamental relationships governing concrete strength, as captured in the S1 dataset, are partially applicable to the S2 dataset, providing a solid foundation for knowledge transfer.

To complement the feature-wise comparison, the t-SNE technique was utilized to reduce the dimensionality of both datasets into a two-dimensional space, as shown in Figure 4d. The plot reveals that the S1 and S2 samples form distinct but adjacent clusters. The absence of a hard separation and the relative proximity of the two clusters indicate that they share underlying structural similarities. This proximity reinforces the conclusion from the distribution analysis, confirming that the knowledge learned from the S1 domain is transferable and can effectively serve as a starting point for modeling the S2 domain, thereby justifying the use of a transfer learning approach.

### 3.4. Data Preprocessing

It is pertinent to clarify the scope of the input features for the modeling process. This study focuses on predicting compressive strength based on the compositional data available in the established datasets. Consequently, physical characteristics of the fresh mix, such as its consistency and moisture content, or properties of the hardened concrete like porosity, were not considered as direct input variables. These factors are implicitly reflected in the final compressive strength outcomes. Similarly, while practical applications often impose constraints on water-to-cement and superplasticizer- to-cement ratios, the model is designed to learn from the wide range of mix proportions present in the source and target datasets without enforcing these specific production limitations.

This study employs a multi-stage data preprocessing approach, encompassing four key steps: component proportion normalization, feature alignment, datasets partitioning, and data augmentation.

Component proportion normalization was performed based on the principle of material component conservation to resolve scale differences in mix proportions between source domain S1 and target domain S2. The feature parameters, including Cement, Water, Coarse Aggregate, Fine Aggregate, and Age, were proportionally scaled using Equations (3) and (4).

Feature alignment was achieved through feature expansion and deletion. For instance, to align the source domain (S1) with the target domain (S2), three new feature columns—Phosphogypsum, PGA, and Retarder—were added to S1, with their corresponding values filled with zeros. This approach was deliberately chosen based on the physical meaning of the features in concrete mix design. A “missing” component, such as Phosphogypsum in a conventional concrete sample, signifies its physical absence in the formulation; therefore, its actual dosage is precisely zero. In this context, zero-filling is not a statistical imputation of a missing value but a direct and accurate representation of the material’s composition.

Alternative methods, such as mean-imputation, were considered inappropriate. Imputing a non-zero mean value for a component that was never present would create a physically unrealistic sample, introducing significant noise and systemic bias that misleads the model. Furthermore, while a missing indicator (mask) is a valid technique, it is largely redundant for tree-based models like LightGBM used in this study. Such models can inherently learn to create decision rules based on a feature’s value being exactly zero (e.g., “if Phosphogypsum <= 0”), effectively capturing the distinction between the presence and absence of a component. Thus, the zero-filling strategy is the most direct, physically meaningful, and algorithmically sound method for aligning the feature spaces.

Concurrently, unique features in S1 not present in S2, such as Blast Furnace Slag and Fly Ash, were removed to ensure the feature columns in both domains were identical. The same alignment methodology was applied when constructing the transfer learning model for the external validation set S3.

To ensure the reproducibility of our results and prevent any information leakage, all stochastic processes, including data partitioning and augmentation, were executed using a fixed random seed (RANDOM_SEED = 42). The data processing protocol was strictly sequenced as follows:

First, for datasets partitioning, all data from the source domain S1 (N = 1030) were designated as part of the joint training set. The target domain data S2 (N = 19) was meticulously split to prevent any data from the test set influencing the training process. It was partitioned into a training set (60%, approx. 11 samples), a validation set (20%, approx. 4 samples), and a test set (20%, approx. 4 samples). The test set was strictly held out and used only for the final, unbiased evaluation of the model’s generalization performance.

Second, for data augmentation, the Bootstrap Resampling technique was applied exclusively to the newly created target domain training set (the 60% portion). This step expanded the target domain training samples to four times their original size (N = 44), resolving the issue of insufficient sample size. This augmented data was then combined with the source domain data (S1) to form the final joint training dataset. This sequential protocol guarantees that the model was trained without any exposure to the test data, ensuring a rigorous and fair evaluation.

### 3.5. Comparative Experimental Design

Four comparative models were designed to systematically evaluate the performance of the proposed transfer learning model in small-sample scenarios: Source Domain Model (SDM), Baseline Model (BLM), Transfer Learning Model (TLM), and Verification Model (VFM). The experiments uniformly adopted Root Mean Square Error (RMSE), Coefficient of determination (R^2^), and Mean Absolute Percentage Error (MAPE) as evaluation metrics to comprehensively assess the models’ predictive performance.

(1) Source Domain Model (SDM)

The SDM model was trained on the source domain S1 (N = 1030). The model’s evaluation metrics, feature importance ranking, SHAP dependency plots, and predicted vs. actual value comparisons were output. This experiment aimed to establish a general mapping relationship between conventional concrete raw materials and compressive strength, identify key parameters influencing strength, and provide prior knowledge for subsequent transfer learning.

(2) Baseline Model (BLM)

For the target domain S2 (N = 19), experiments were conducted using RF and XGBoost algorithms. During the experiments, a randomly partitioned training dataset (80%) was used for model training, and the final performance was evaluated on the test datasets (20%). Notably, due to the extremely small sample size of S2 (only 15 training samples), the LightGBM algorithm failed to converge because of gradient boosting iteration failure and was thus excluded from the baseline comparison. This experiment aimed to quantify the performance of traditional methods under small-sample conditions and establish a benchmark for comparison with the transfer learning model.

(3) Transfer Learning Model (TLM)

Based on the dynamic weighted transfer learning framework, the source domain S1 (N = 1030) and the augmented target domain S2 training set (N = 44) were jointly trained. The error feedback strategy dynamically adjusted the weights of target domain samples, prioritizing the learning of prediction-blind samples in the target domain. The experiment simultaneously output evaluation metrics, feature importance distributions, and SHAP dependency plots to analyze the contribution of unique components such as Phosphogypsum and Retarder. This experiment validated the effectiveness of the dynamic weighted transfer learning model in knowledge transfer.

(4) Verification Model (VFM)

The trained S1→S2 transfer learning model framework was applied to the external validation datasets S3 (N = 18) to test its predictive performance under reduced feature dimensionality and small-sample conditions. By comparing the results of the transfer learning model with traditional machine learning models, the model’s generalization capability on external small-sample datasets was evaluated, providing reliable validation for similar PGC strength prediction tasks. It is important to emphasize that the S3 dataset was strictly held out as an independent test set. It was not used in any part of the model training, validation, or hyperparameter tuning processes, ensuring an unbiased evaluation of the final model’s generalization performance.

A repeated random sub-sampling validation strategy was employed instead of a single data split to ensure the robustness and reliability of the model evaluation. The target domain dataset was subjected to 10 independent iterations. In each iteration, the data was randomly partitioned into a training set, a validation set, and a test set. The data augmentation and model training process were performed anew for each iteration. The final performance metrics—RMSE, R^2^, and MAPE—were calculated by averaging the results from all 10 test sets. The standard deviation (SD) was also computed to quantify the uncertainty and stability of the models’ predictive performance across different data partitions. This rigorous approach mitigates potential biases from any single data split and provides a more comprehensive assessment of the models’ generalization capabilities.

## 4. Results and Discussion

### 4.1. Results of the Source Domain Model (SDM)

The SDM model demonstrated strong performance in predicting the compressive strength of conventional concrete. On the test set, the model achieved R^2^ = 0.95, RMSE = 3.81 MPa, and MAPE = 9.05%, indicating its ability to accurately capture the complex nonlinear relationships between feature parameters and strength.

**Figure 5:**
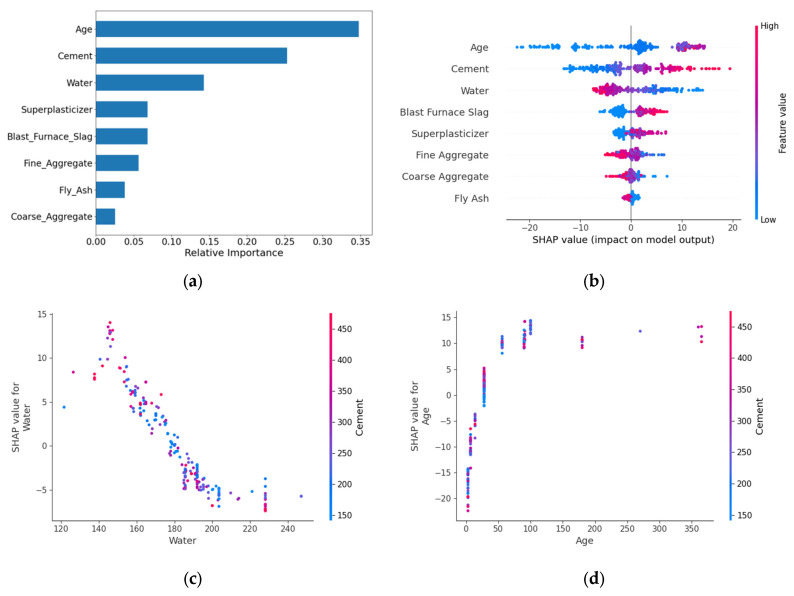
Feature importance analysis and SHAP dependence of the SDM. (**a**) Feature importance; (**b**) SHAP overview; (**c**) Water-Cement dependency; (**d**) Age-Cement dependency.

The feature importance analysis (Figure 5) revealed that Age, Cement, and Water constituted the core control variables, with a cumulative contribution of 74.4%. The remaining parameters were ranked in descending order of importance as follows: Superplasticizer, Blast Furnace Slag, Fine Aggregate, Fly Ash, and Coarse Aggregate. The SHAP overview plots showed that Age and Cement exhibited the widest distribution of positive SHAP values, indicating a significant positive correlation with strength. In contrast, Water displayed a negative effect, aligning with the water-cement ratio theory. High feature values (red) for Age and Cement were concentrated on the right side, while high Water values were clustered on the left, consistent with the hydration reaction mechanism in material science. Other features, such as Blast Furnace Slag, Superplasticizer, Fine Aggregate, Coarse Aggregate, and Fly Ash, had relatively minor impacts on model output. The Water-Cement SHAP dependence plot reveals that samples with higher Cement content (red-colored) exhibit lower SHAP values at elevated Water content, further corroborating the negative impact of Water content on model outputs. The Age-Cement SHAP dependence plot demonstrates an exponential relationship between Age and Strength development, characterized by an initially steep increase followed by gradual stabilization. Color gradient analysis indicates no significant correlation between the combined effects of Age and Cement on compressive strength.

**Figure 6:**
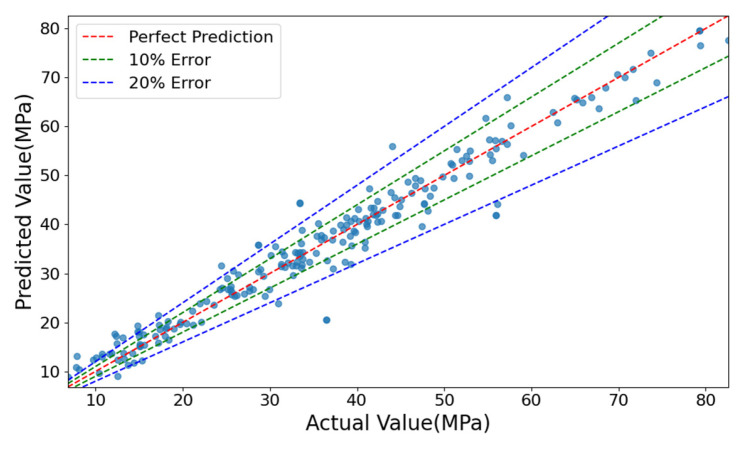
Comparison between predicted values and actual values of the SDM.

Figure 6 compares the model’s predicted values with the actual values on the test datasets. Most data points lie close to the ideal prediction line, indicating strong predictive accuracy. However, some deviations occur in the low-strength region, where a few predictions fall outside the ±20% error band. Nevertheless, over 70% of the data points remain within the ±10% error range, meeting the expected accuracy requirements.

### 4.2. Results of the Baseline Model (BLM)

As shown in Table 4, the BLM trained on the target domain datasets S2, XGBoost, and RF was evaluated 0.81, and MAPE of 7.70%, while the RF model performed slightly better with an RMSE of 3.20 MPa, R^2^ of 0.83, but a marginally higher MAPE of 9.21%.

**Figure 7:**
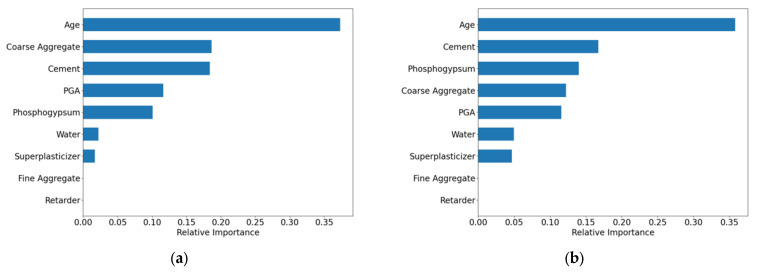
Feature importance results of the BLM. (**a**) XGBoost; (**b**) RF.

As illustrated in Figure 7, the feature importance analysis showed both similarities and differences between the two models. Both identified Age as the most critical feature (importance > 0.35), significantly outweighing other variables. However, the secondary feature rankings diverged:

XGBoost: Coarse Aggregate > Cement > PGA > Phosphogypsum > Water > Superplasticizer > Fine Aggregate > Retarder.

RF: Cement > Phosphogypsum > Coarse Aggregate > PGA > Water > Superplasticizer > Fine Aggregate > Retarder.

This discrepancy arises from the limited sample size (N = 19), which introduces instability in feature importance estimation under small-sample conditions. Notably, both models assigned zero importance to Fine Aggregate and Retarder, mainly due to their low data variability, making it difficult to discern their relationship with compressive strength.

**Figure 8:**
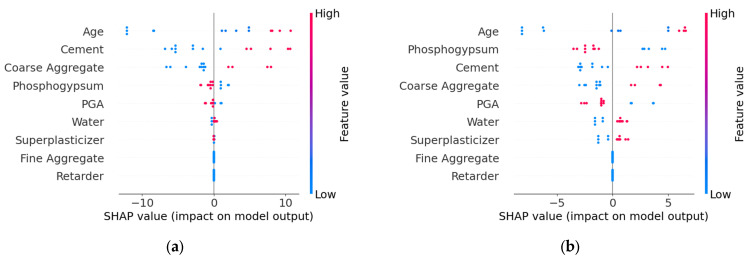
Comparison of SHAP values. (**a**) XGBoost; (**b**) RF.

The SHAP overview shown in Figure 8 indicates that different algorithms exhibit variations in feature interpretation. In the XGBoost model, Age, Cement, and Coarse Aggregate have the greatest impact on the model’s output, with relatively dispersed SHAP value distributions, suggesting these features contribute significantly to the model’s predictions. In contrast, the RF model shows more pronounced influences from Age, Phosphogypsum, and Cement. Additionally, Fine Aggregate and Retarder display minimal SHAP value variations in both XGBoost and RF models, indicating that both algorithms consider their impact on strength to be negligible. The SHAP values of other parameters calculated by the two models differ in both ranking and distribution, demonstrating clear disparities.

The above analysis reveals that even when using the same datasets, the output results can vary significantly depending on the machine learning algorithm employed. This distinction is particularly prominent in small-sample data. The primary reason is that in small samples, the true relationships between features are difficult to capture, leading the models to misinterpret random fluctuations as meaningful patterns.

**Figure 9:**
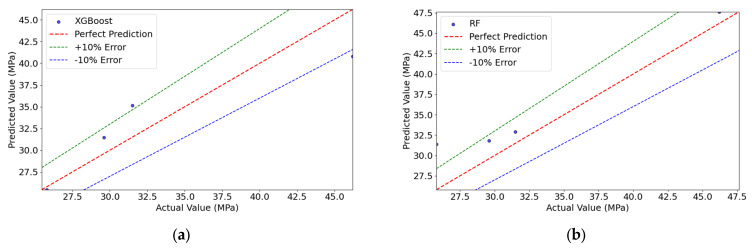
Comparison of actual and predicted values in the BLM. (**a**) XGBoost; (**b**) RF.

Figure 9 compares the predicted and actual values for both models. RF demonstrated better fitting in the medium-to-high strength range, while XGBoost performed well in the low-to-medium range. Overall, most predictions fell within the ±10% error band, confirming reasonable accuracy.

In the S2 target domain datasets, the predictive capability of traditional machine learning models is constrained by the limited sample. Although the XGBoost and RF models achieve an average R^2^ score of 0.82 on the test set, demonstrating basic predictive performance, this remains insufficient to meet engineering-level strength prediction requirements. Furthermore, feature importance analysis exhibits significant randomness, reflecting the instability in feature contribution interpretation due to the small sample. SHAP value comparisons reveal that the models struggle to capture stable correlation mechanisms among components from the limited data.

### 4.3. Results of the Transfer Learning Model (TLM)

Table 5 presents the test results of the TLM, constructed using the dynamic weighted transfer learning framework. Compared to the average performance of the BLM, the TLM demonstrated superior and more stable results. The RMSE was reduced from 3.31 MPa to 2.86 MPa, representing a 13.6% decrease. The R^2^ significantly improved from 0.82 to 0.95, a 15.9% increase, and the MAPE decreased from 8.48 to 7.78. These results validate the effectiveness of the dynamic weighted transfer learning model in small-sample conditions, demonstrating its ability to overcome the performance limitations of traditional methods under extreme data scarcity conditions.

**Figure 10:**
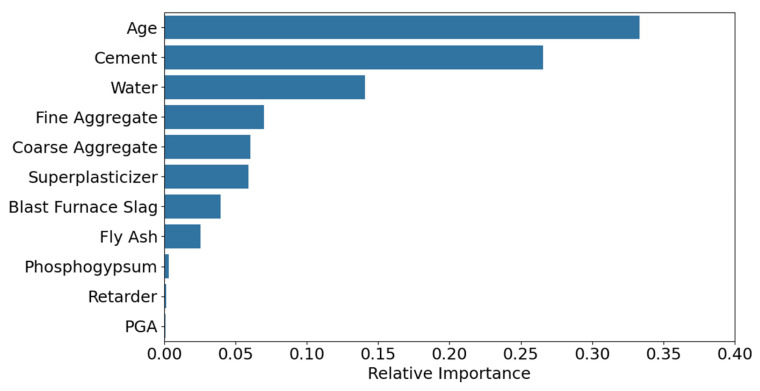
Feature importance of the TLM.

Figure 10 displays the feature importance distribution output by the TLM. The feature Age has the highest importance score (≈0.35), indicating its critical role in model predictions, followed by Cement and Water, with importance scores of 0.27 and 0.14, respectively. Other secondary features are ranked as follows: Fine Aggregate, Coarse Aggregate, Superplasticizer, Blast Furnace Slag, Fly Ash, Phosphogypsum, Retarder, and PGA. Compared to the feature importance results of the SDM, both models identify Age, Cement, and Water as the top three most important features, confirming that TLM successfully inherits the general knowledge of conventional concrete. Notably, the target domain-specific features (Phosphogypsum, Retarder, and PGA) exhibit low but non-zero importance scores, indicating that TLM captures their unique influence on strength.

**Figure 11:**
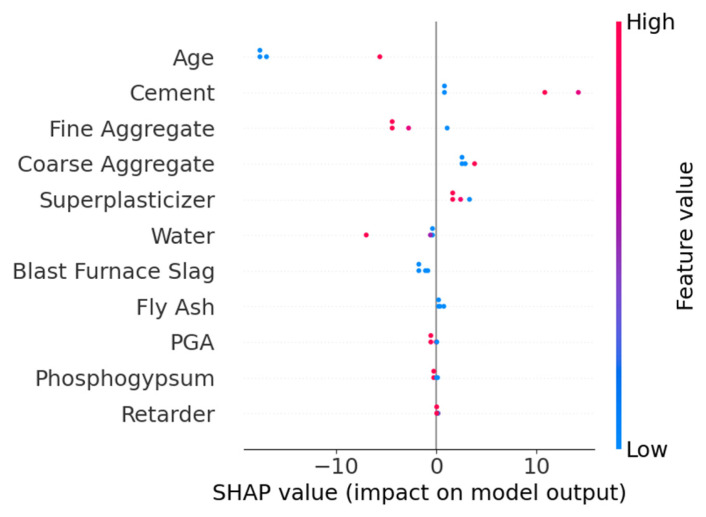
SHAP overview of the TLM model.

Figure 11 shows the SHAP overview for feature contributions. The features Age and Cement exhibit the most significant impact on model outputs. Specifically, lower values of Age negatively affect strength, while higher values of Cement positively correlate with strength. The SHAP values of other features are more concentrated, suggesting their relatively minor influence on strength predictions.

A sensitivity analysis was conducted to assess the stability and robustness of the SHAP attributions. The proposed TLM was retrained 10 times, each with a different random seed, to account for stochastic variability during the training process. For each of the 10 runs, SHAP values were computed on the test set. The mean and standard deviation of the absolute SHAP values for each feature were then calculated across all runs to quantify the variance.

The results are presented in Table 6. The standard deviations for all feature attributions are consistently low relative to their mean values, particularly for the most influential features such as Age, Cement, and Fine Aggregate. This low variance indicates that the feature importance and attributions generated by the model are stable and not highly sensitive to model reruns. This robustness strengthens the reliability of the conclusions drawn from the SHAP analysis regarding the underlying mechanisms of PGC strength development.

**Figure 12:**
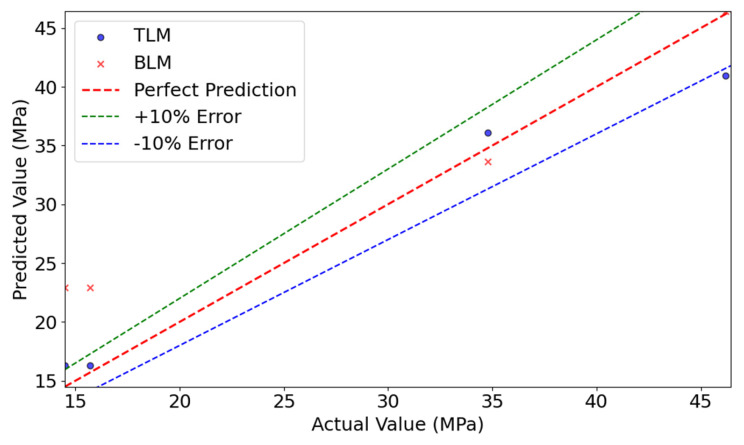
Comparison of actual and predicted values between TLM and BLM.

Figure 12 compares the prediction results of the TLM and BLM. The plot illustrates a representative run where the TLM achieved the median R^2^ score among the 10 iterations, while the aggregated statistical performance is presented in Table 5. The TLM model’s predictions closely align with the true values, with most data points distributed near the ideal prediction line, indicating superior predictive performance. In contrast, the BLM model shows larger deviations, particularly in low-value regions, where predictions significantly diverge from the ideal line. This analysis confirms that the TLM outperforms the BLM in both prediction accuracy and generalization capability.

In summary, the proposed dynamic weighted transfer learning framework achieves high-precision strength prediction for PGC, effectively addressing the challenges posed by limited sample sizes and cross-material domain knowledge transfer.

### 4.4. Results of the Verification Model (VFM)

As shown in Table 7, the performance of the VFM model on the external validation datasets (S3) was evaluated. When evaluated on the external validation datasets (S3) using the same repeated random splits methodology, the BLM (XGBoost) achieved a RMSE of 11.47 MPa, R^2^ of 0.86, and MAPE of 13.80. In stark contrast, the VFM model demonstrated significantly improved and more stable performance, with a mean RMSE of 3.40 MPa, R^2^ of 0.97, and MAPE of 5.30%. This comparison highlights the performance degradation of traditional methods when faced with reduced feature dimensions and material system differences. In contrast, the TLM, through dynamic weight calculation and weighted loss optimization, achieved highly accurate strength predictions for PGC.

To provide a more granular view of the model’s performance on the external dataset, Table 8 details the per-sample prediction results for the S3 dataset. The table shows that the model maintains consistently small residuals across the entire range of strength values, further validating its high accuracy and generalization capability.

**Figure 13:**
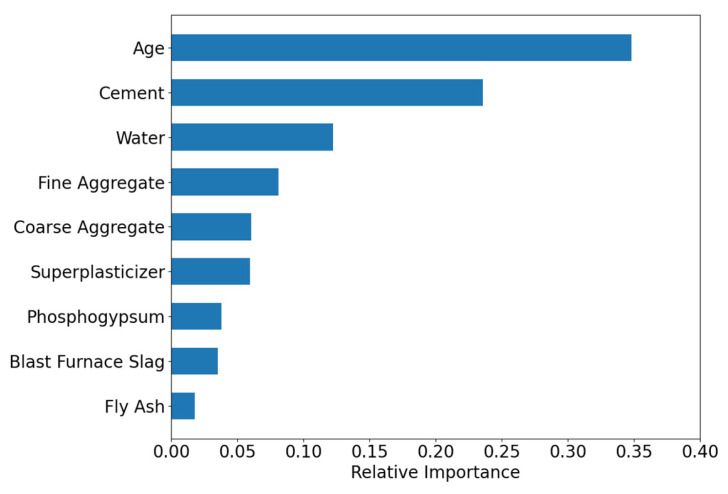
Feature importance of the VFM.

Figure 13 presents the feature importance ranking computed by the VFM model. Similar to the SDM model, Age has the highest importance score (0.35), followed by Cement (0.25) and Water (0.13), indicating that the transfer learning model successfully captured the general strength prediction rules of conventional concrete. Other features, such as Fine Aggregate (0.08), Coarse Aggregate (0.07), and Superplasticizer (0.06), had relatively lower importance scores, suggesting their limited contribution to model predictions. Features like Phosphogypsum (0.04), Blast Furnace Slag (0.03), and Fly Ash (0.02) exhibited even weaker influence. Importantly, all features had non-zero importance scores, confirming that the transfer learning model successfully identified their impact on strength.

**Figure 14:**
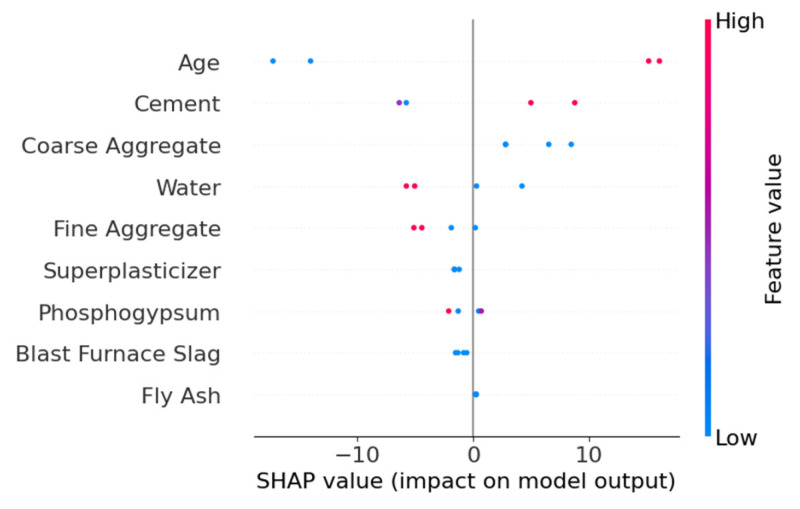
SHAP overview plot of the VFM.

Figure 14 illustrates the SHAP overview for feature contributions. The Age and Cement features show dispersed SHAP values, indicating their complex influence on model outputs (both positive and negative effects). In contrast, Coarse Aggregate, Water, and Fine Aggregate exhibit more concentrated SHAP values, suggesting a more consistent impact. Other features, such as Superplasticizer, Phosphogypsum, Blast Furnace Slag, and Fly Ash, had minimal influence on strength predictions.

**Figure 15:**
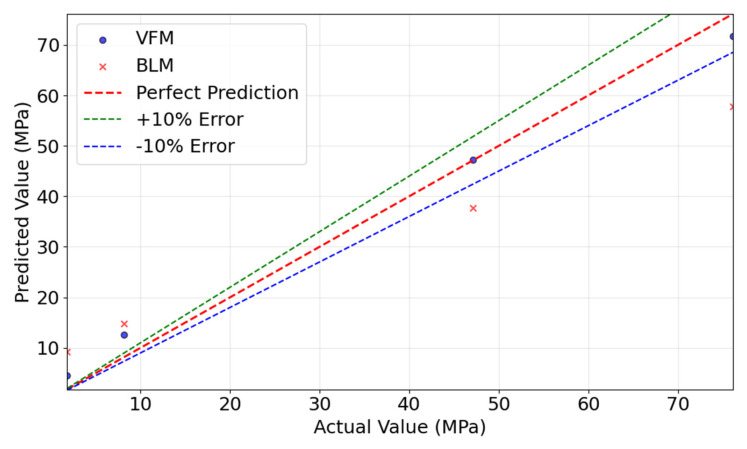
Comparison of actual and predicted values between VFM and BLM.

Figure 15 compares the predicted and actual values for the VFM and BLM models. The VFM model’s predictions closely follow the ideal prediction line, whereas the baseline model shows significant deviations, particularly in high-strength regions. This further confirms the superior predictive performance of the transfer learning model.

The proposed transfer learning model demonstrates excellent predictive performance on external test sets, making it applicable to similar PGC strength prediction tasks.

### 4.5. Discussion

(1) Comparative analysis of transfer learning model adaptability across different algorithms

**Figure 16:**
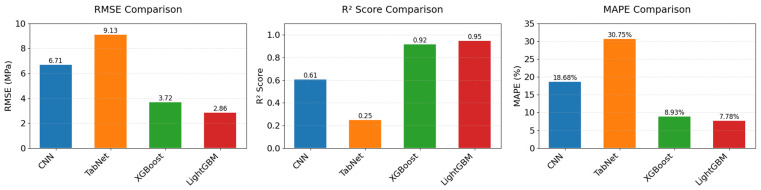
Performance comparison of different transfer learning algorithms.

To evaluate how algorithmic adaptability affects cross-domain generalization in transfer learning, this study implements CNN-, TabNet-, and XGBoost-based models for systematic comparison with LightGBM. The experimental results are shown in Figure 16, with detailed data in Table 9. The experiments revealed significant performance differences among algorithms in cross-domain transfer scenarios. The CNN model achieved an RMSE of 6.71 MPa, R^2^ of 0.61, and MAPE of 18.68% on the test datasets, with its prediction accuracy notably lower than that of tree-based ensemble models. This is primarily because CNN’s convolutional inductive bias is more suited for localized correlation data (e.g., images), whereas the global nonlinear interactions among concrete mix features are difficult to capture effectively via convolutional kernels, especially when sample sizes are limited, leading to suboptimal parameter optimization.

The TabNet model triggered early stopping at the 88th iteration, with severely degraded test performance (RMSE = 9.13 MPa, R^2^ = 0.25). This indicates that its attention-based sequential modeling strategy suffers from overfitting under extremely small sample conditions, suggesting the model merely memorized noise in the training data rather than learning the underlying physical laws. In contrast, the XGBoost model achieved strong results (RMSE = 3.72, R^2^ = 0.92), approaching the performance of the LightGBM model (R^2^ = 0.95). This confirms the universal advantage of gradient-boosted tree algorithms in material data modeling. The performance gap between the two mainly stems from LightGBM’s histogram acceleration and dynamic weight allocation strategy. The histogram algorithm reduces sensitivity to small-sample noise through feature discretization, while the error-feedback weighting strategy allows the model to focus on high-residual spots, making it more adaptable to the local nonlinear characteristics of PGC compared to XGBoost’s global loss optimization. These results demonstrate that tree-based ensemble algorithms exhibit stronger adaptability in cross-domain transfer tasks, whereas neural network models require sufficient sample sizes to overcome the curse of dimensionality.

(2) Hyperparameter sensitivity analysis for dynamic weights

**Figure 17:**
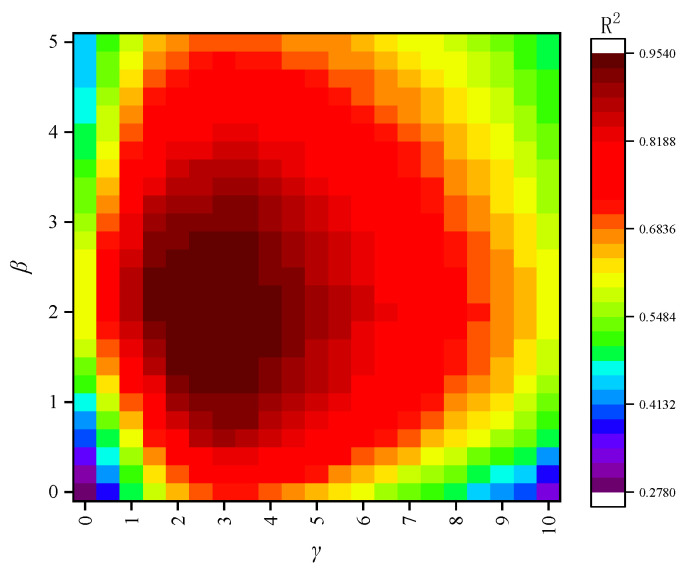
Heatmap of R^2^ scores on the validation set for different combinations of hyperparameters *β* and *γ*.

The hyperparameters *β* and *γ* in the dynamic weighting formula (Equation (6)) are crucial as they control the baseline importance and the dynamic adjustment range for the target domain samples. To justify the selection of *β* = 2 and *γ* = 3, a grid search-based sensitivity analysis was conducted. We systematically varied *β* within the range of [0, 5] and *γ* within the range of [0, 10], training the transfer learning model for each combination and evaluating its performance on the validation set. The results are visualized as a heatmap of the R^2^ score in Figure 17.

The heatmap reveals that the model’s performance is highly dependent on the choice of these parameters. A clear optimal region emerges where R^2^ is maximized. The parameter *β*, which sets the minimum weight for target domain samples relative to source domain samples, shows poor performance when close to 0. This is because a very low base weight can make the model unstable and fail to give sufficient priority to the target domain. As *β* increases, performance improves, but excessively high values (e.g., >4) lead to diminishing returns, as the model starts to ignore the nuanced error feedback provided by *γ*. The parameter *γ*, which scales the importance of high-error samples, demonstrates that a complete lack of dynamic adjustment results in suboptimal performance. As *γ* increases, the model’s ability to focus on difficult samples improves performance. However, if *γ* is too large, the model becomes susceptible to overfitting to potential outliers in the small training set, causing a slight degradation in generalization performance.

The combination of *β* = 2 and *γ* = 3 is situated squarely within the high-performance plateau of the metric surface. This choice ensures that all target domain samples receive at least double the weight of source domain samples, while also providing a significant but stable dynamic range for error-based adjustments. This balanced approach was therefore adopted for the final model.

(3) Applicability boundary analysis for small-sample target domains

**Figure 18:**
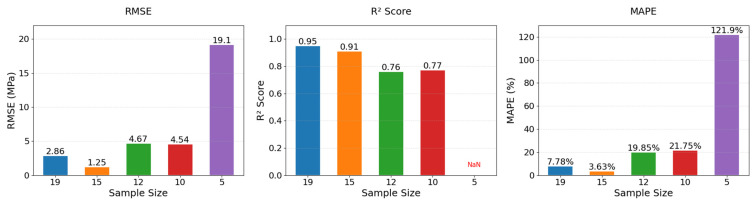
Comparison of model performance under different sample sizes.

Through sensitivity analyses involving incremental reductions in the number of training samples in the target domain, this study quantitatively establishes the minimum viable sample threshold for transfer learning models, empirically mapping the performance degradation frontier in small-sample regimes. Sample sizes were set to *N* = 15, 12, 10, and 5, with results compared in Figure 18 and metrics listed in Table 10.

When the sample size was reduced to 15, the model maintained high predictive performance (RMSE = 1.25 MPa, R^2^ = 0.91, MAPE = 3.63%), with R^2^ only 4.2% lower than the full sample datasets (*N* = 19), indicating reliable prediction capability for sample sizes ≥15. However, when the sample size dropped to 12, performance deteriorated sharply (RMSE = 4.67 MPa, R^2^ = 0.76, MAPE = 19.85%), with R^2^ decreasing by 20.0%, suggesting the model began losing stable mapping ability between material components and strength. Further reduction to 10 samples resulted in R^2^ = 0.77 but worsened MAPE to 21.75%, indicating extreme prediction biases for some samples. At *N* = 5, the model completely failed (R^2^ = NaN, MAPE = 121.92%), with predictions showing random correlations to true values. These analyses demonstrate that 15 samples represent the minimum critical threshold for maintaining the stability of the transfer learning in PGC strength prediction.

(4) Analysis of data augmentation multiplier

**Figure 19:**
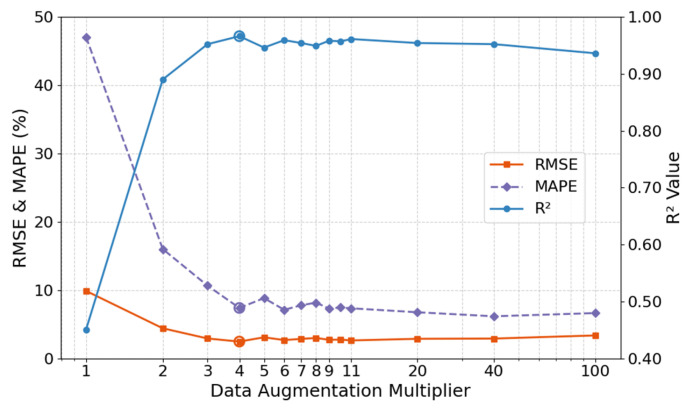
Analysis of data augmentation multiplier.

The data augmentation multiplier governs the target-domain knowledge supplementation intensity in transfer learning, representing a critical trade-off parameter between generation efficiency and model generalization capability. This study systematically tested prediction performance across augmentation multipliers ranging from 1 to 100×, quantitatively revealing the nonlinear relationship between data expansion scale and model accuracy. The results in Figure 19 showed a non-monotonic relationship between augmentation multiplier and model performance, divisible into two phases:

(a) Rapid optimization phase (1~4×): RMSE plummeted from 9.87 MPa to 2.47 MPa, R^2^ surged from 0.45 to 0.95, and MAPE shrank from 47.03% to 7.78%. Bootstrap Resampling effectively mitigated underfitting caused by insufficient original samples, with augmented samples significantly enhancing predictive capability.

(b) Stabilization phase (5~100×): Performance plateaued, with RMSE fluctuations < 0.8 MPa and R^2^ maintained at 0.94~0.96, though marginally lower than the 4× peak. Thus, a 4× augmentation multiplier is recommended to ensure effective small-sample expansion while avoiding overfitting risks.

(5) Analysis of physical plausibility

To validate the model’s adherence to fundamental material science principles, a sensitivity analysis was conducted to examine the relationship between cement content and predicted compressive strength. In this analysis, the water-cement ratio was held constant at 0.45. All other parameters were held constant, with values set as follows: Superplasticizer = 12.0 kg/m^3^, Fine Aggregate = 810.0 kg/m^3^, PGA = 510.0 kg/m^3^, Retarder = 1.2 kg/m^3^, Phosphogypsum = 299.8 kg/m^3^, and Age = 28 days. The cement content was then incrementally increased, and the corresponding strength predictions from the trained TLM model were recorded.

The results, presented in Table 11, demonstrate a clear and positive trend. As the cement content increases from 300 kg/m^3^ to 550 kg/m^3^, the predicted compressive strength generally rises from 48.7 MPa to 53.9 MPa. This outcome aligns with the established physical law that, within a certain range, higher cement content leads to greater strength. Notably, the model revealed a slight non-monotonic dip, a behavior reflecting its data-driven nature. Unlike models with monotonic constraints, it captures complex non-linear interactions from the data. This flexibility allows it to learn nuanced relationships while respecting general physical laws, confirming its predictions are physically sound and robust. This analysis confirms that the model successfully captured this fundamental monotonic relationship without being explicitly constrained. The ability of the TLM to learn such physical laws, even from limited target domain data, underscores the effectiveness of the knowledge transfer from the large, diverse source domain, enhancing its reliability for practical engineering applications.

## 5. Conclusions

This study addresses the critical challenges of small sample sizes and cross-domain knowledge transfer in predicting the compressive strength of PGC by constructing a dynamic weighted transfer learning framework. By integrating large-sample data from conventional concrete (source domain) with small-sample data from PGC (target domain), a high-precision predictive model for industrial solid waste-based PGC was developed. The main conclusions are as follows:

(1) The proposed dynamic weighted transfer learning framework effectively mitigates the underfitting issue of machine learning models under small-sample conditions. By eliminating feature disparities between conventional concrete and PGC through component proportion normalization and feature alignment, combined with an error feedback-driven dynamic weight calculation and weighted loss optimization strategy, the coefficient of determination R^2^ on the target domain test datasets reached 0.95, representing a 15.9% improvement over traditional methods. The external validation set maintained a high-precision R^2^ of 0.97.

(2) The Bootstrap Resampling data augmentation method overcomes the limitations of traditional oversampling techniques. By generating samples that adhere to the conservation laws of mix proportions, the target domain datasets were effectively expanded while preserving material physicochemical properties, resolving the underfitting problem under small-sample conditions.

(3) The transfer learning framework successfully achieved cross-material domain knowledge transfer and stable feature interpretation. The model learned general knowledge from large-sample conventional concrete data in the source domain. After transfer to the target domain, it not only maintained stable interpretation of shared features but also accurately captured the nonlinear effects of PGC-specific components on strength.

(4) Comparative experiments on algorithms demonstrated the superiority of tree-based ensemble methods in transfer learning tasks. Applicability analysis revealed that the model maintains high predictive reliability when the target domain sample size was ≥15. Systematic analysis of data augmentation multiples revealed that quadrupling the target domain training datasets size produced optimal model performance, beyond which further augmentation resulted in performance saturation or marginal deterioration.

The dynamic weighted transfer learning framework developed in this study provides an innovative approach for the intelligent design of industrial solid waste-based PGC. The applicability of this framework is not limited to PGC but extends to other technical challenges characterized by small-sample constraints in materials science. For instance, it can be adapted to predict the performance of concrete incorporating other industrial byproducts, such as steel slag or silica fume, or to forecast other critical properties like elastic modulus and durability.

Building on this foundation, several avenues for future research are proposed. First, future work should rigorously validate the framework’s generalizability across a wider range of multi-material systems. Second, a pivotal direction is the development of material science-based sample screening mechanisms. This would enable the optimization of data partitioning strategies guided by physicochemical constraints, moving beyond random sampling to reduce bias and significantly enhance model robustness. Finally, a promising long-term goal is to leverage this predictive model for inverse material design, creating an intelligent system capable of autonomously formulating concrete mixtures to meet specific, predefined performance targets.

## Figures and Tables

**Table 1:** Distribution of source domain datasets S1.

Statistical Metric	Min.	Max.	Mean	Median	Std. Dev.
Cement (kg/m^3^)	102.0	540.0	281.2	272.9	104.5
Blast Furnace Slag (kg/m^3^)	0.0	359.4	73.9	22.0	86.3
Fly Ash (kg/m^3^)	0.0	200.1	54.2	0.0	64.0
Water (kg/m^3^)	121.8	247.0	181.6	185.0	21.4
Superplasticizer (kg/m^3^)	0.0	32.2	6.2	6.3	6.0
Coarse Aggregate (kg/m^3^)	801.0	1145.0	972.9	968.0	77.8
Fine Aggregate (kg/m^3^)	594.0	992.6	773.6	779.5	80.2
Age (day)	1.0	365.0	45.7	28.0	63.2
Compressive Strength (MPa)	2.3	82.6	35.8	34.4	16.7

**Table 2:** Distribution of target domain datasets S2.

Statistical Metric	Min.	Max.	Mean	Median	Std. Dev.
Cement (kg/m^3^)	299.8	608.4	404.1	304.2	148.6
Water (kg/m^3^)	156.0	206.9	189.9	206.9	24.7
Superplasticizer (kg/m^3^)	12.0	12.2	12.1	12.2	0.1
Coarse Aggregate (kg/m^3^)	0.0	810.0	270.0	0.0	392.9
Fine Aggregate (kg/m^3^)	810.0	810.0	810.0	810.0	0.0
PGA (kg/m^3^)	0.0	510.0	340.0	510.0	247.4
Retarder (kg/m^3^)	1.2	1.2	1.2	1.2	0.0
Phosphogypsum (kg/m^3^)	0.0	304.2	201.3	299.8	146.5
Age (day)	3	28	10.1	7	10.0
Compressive Strength (MPa)	14.5	68.3	34.3	33.2	13.9

**Table 3:** Distribution of external validation datasets S3.

Statistical Metric	Min.	Max.	Mean	Median	Std. Dev.
Cement (kg/m^3^)	45.0	450.0	262.5	270.0	147.4
Water (kg/m^3^)	225.0	225.0	225.0	225.0	0.0
Fine Aggregate (kg/m^3^)	1350.0	1350.0	1350.0	1350.0	0.0
Phosphogypsum (kg/m^3^)	0.0	405.0	187.5	180.0	147.4
Age (day)	3.0	28.0	12.7	7.0	11.3
Compressive Strength (MPa)	0.6	95.0	33.0	24.6	29.8

**Table 4:** Performance of BLM.

Model	RMSE (MPa)	R^2^	MAPE (%)
XGBoost	3.41 ± 0.25	0.81 ± 0.04	7.75 ± 0.95
RF	3.20 ± 0.21	0.83 ± 0.03	9.21 ± 1.10
Average	3.31 ± 0.23	0.82 ± 0.04	8.48 ± 1.03

Note: Results are reported as mean ± standard deviation over 100 random splits.

**Table 5:** Performance comparison between TLM and BLM.

Model	RMSE (MPa)	R^2^	MAPE (%)	Training Sample Size
BLM	3.31 ± 0.23	0.82 ± 0.04	8.48 ± 1.03	11
TLM	2.86 ± 0.18	0.95 ± 0.02	7.78 ± 0.85	1074 (1030 + 44)

Note: Results are reported as mean ± standard deviation over 100 random splits.

**Table 6:** Stability analysis of mean absolute SHAP values for the TLM across 10 reruns.

Feature	Mean Absolute SHAP Value	Standard Deviation
Age	10.34	2.56
Cement	6.60	1.25
Fine Aggregate	3.97	0.89
Coarse Aggregate	2.73	0.78
Superplasticizer	2.21	0.75
Water	2.10	0.62
Blast Furnace Slag	1.60	0.27
Fly Ash	0.68	0.08
PGA	0.48	0.03
Phosphogypsum	0.21	0.02
Retarder	0.05	0.01

**Table 7:** Performance comparison between VFM and BLM.

Model	RMSE (MPa)	R^2^	MAPE (%)
BLM	11.47 ± 0.95	0.86 ± 0.05	13.80 ± 1.55
VFM	3.40 ± 0.28	0.97 ± 0.01	5.30 ± 0.60

**Table 8:** Per-sample prediction results of the VFM on the external validation set S3.

Sample No.	Actual Strength (MPa)	Predicted Strength (MPa)	Residual (MPa)
1	76.16	71.72	4.44
2	8.17	12.55	−4.37
3	1.78	4.52	−2.74
4	47.13	47.23	−0.10

**Table 9:** Performance comparison of transfer learning models with different algorithms.

Transfer Learning Algorithm	RMSE (MPa)	R^2^	MAPE
CNN	6.71	0.61	18.68%
TabNet	9.13	0.25	30.75%
XGBoost	3.72	0.92	8.93%
LightGBM (Proposed)	2.86	0.95	7.78%

**Table 10:** Model performance metrics under different sample sizes.

Sample Size	RMSE (MPa)	R^2^	MAPE
19	2.86	0.95	7.78%
15	1.25	0.91	3.63%
12	4.67	0.76	19.85%
10	4.54	0.77	21.75%
5	19.14	NaN	121.92%

**Table 11:** Predicted compressive strength for sensitivity analysis.

Cement (kg/m^3^)	Water (kg/m^3^)	Water-Cement Ratio	Predicted Compressive Strength (MPa)
300	135.0	0.45	48.7
350	157.5	0.45	49.3
400	180.0	0.45	51.4
450	202.5	0.45	50.3
500	225.0	0.45	52.9
550	247.5	0.45	53.9

## Data Availability

The raw data supporting the conclusions of this article will be made available by the authors on request.
